# Investigating the Impact of Glycogen-Depleting Exercise Combined with Prolonged Fasting on Autophagy and Cellular Health in Humans: A Randomised Controlled Crossover Trial

**DOI:** 10.3390/nu16244297

**Published:** 2024-12-12

**Authors:** Andrius Masedunskas, Isabella de Ciutiis, Leanne K. Hein, Anjie Ge, Yvonne X. Kong, Miao Qi, Drishya Mainali, Lara Rogerson-Wood, Cynthia M. Kroeger, Yvonne A. Aguirre Candia, Maria L. Cagigas, Tian Wang, David Hutchinson, Angelo Sabag, Freda H. Passam, Laura Piccio, Timothy J. Sargeant, Luigi Fontana

**Affiliations:** 1 Charles Perkins Centre, University of Sydney, Sydney, NSW 2006, Australia; andrius.masedunskas@sydney.edu.au (A.M.); anjie.ge@sydney.edu.au (A.G.); angelo.sabag@sydney.edu.au (A.S.); freda.passam@sydney.edu.au (F.H.P.);; 2 Central Clinical School, Faculty of Medicine and Health, University of Sydney, Sydney, NSW 2006, Australia; 3 Lysosomal Health in Ageing, Lifelong Health Theme, South Australian Health and Medical Research Institute (SAHMRI), Adelaide, SA 5000, Australia; 4 Department of Endocrinology, Royal Prince Alfred Hospital, Sydney, NSW 2006, Australia; 5 Faculty of Health and Medical Sciences, The University of Adelaide, Adelaide, SA 5005, Australia

**Keywords:** fasting, exercise training, autophagy, ketone bodies, metabolism

## Abstract

Importance: Although prolonged fasting has become increasingly popular, the favourable biological adaptations and possible adverse effects in humans have yet to be fully elucidated. Objective: To investigate the effects of a three-day water-only fasting, with or without exercise-induced glycogen depletion, on autophagy activation and the molecular pathways involved in cellular damage accumulation and repair in healthy humans. Design: A randomised, single-centre, two-period, two-sequence crossover trial. The primary outcome is autophagic activity, assessed as flux in peripheral blood mononuclear cells (PBMCs) measured in the context of whole blood. Secondary outcomes include changes in body composition, heart rate variability, endothelial function, and genomic, epigenomic, metabolomic, proteomic, and metagenomic adaptations to fasting in plasma, platelets, urine, stools, and PBMCs. Detailed profiling of circulating immune cell populations and their functional states will be assessed by flow cytometry. Setting: All clinical investigations will be undertaken at the Charles Perkins Centre Royal Prince Alfred Hospital clinic, University of Sydney, Australia. Participants: Twenty-four individuals aged 18 to 70 years, with a BMI of 20–40 kg/m^2^, free of major health conditions other than obesity. Discussion: While autophagic flux induction through fasting has garnered interest, there is a notable lack of human studies on this topic. This trial aims to provide the most detailed and integrated analysis of how three days of prolonged water-only fasting, combined with glycogen-depleting exercise, affects autophagy activation and other crucial metabolic and molecular pathways linked to cellular, metabolic, and immune health. Insights from this study may pave the way for safe and effective strategies to induce autophagy, offering potential preventive interventions for a range of chronic conditions.

## 1. Introduction

Prolonged fasting, defined as extended abstinence from calorie intake, lasting longer than 36 or 48 h, has deep roots in religious and cultural traditions across many faiths, including Hinduism, Buddhism, Judaism, Islam, and Christianity [1]. Despite its ancient origins, the first detailed scientific investigations into the physiological effects of prolonged fasting in humans were conducted in the 1960s and 1970s by Cahill and colleagues, long before the advent of molecular biology [2,3,4,5]. Their work demonstrated that fasting triggers profound metabolic adaptations aimed at conserving energy and feeding essential organs with ketones, while minimising the breakdown of muscle protein, enabling survival on minimal resources for extended periods [3]. Remarkably, they found that a person of average weight could survive up to two months on water alone, whereas individuals with obesity are capable of fasting for much longer [6]. One extraordinary case involved a 207 kg man who fasted for 382 days, consuming only calorie-free fluids, vitamins, and minerals, resulting in a dramatic weight loss of 126 kg or 61% of his initial body weight [7].

In recent years, periodic prolonged fasting has gained renewed attention due to claims that it promotes longevity and improves health by influencing cellular ageing and reducing disease risk factors. However, despite its growing popularity, the precise molecular and cellular adaptations and potential risks associated with fasting in humans remain poorly understood [8,9]. For example, one of the key mechanisms linked to fasting’s benefits is autophagy, a tightly regulated process responsible for degrading and recycling damaged organelles and misfolded proteins, thus maintaining cellular homeostasis with ageing [10]. While evidence from animal models supports fasting-induced autophagy, human studies have largely relied on indirect and imprecise markers, such as protein or mRNA levels, as indicators of autophagic activity [11]. The precise dynamics of autophagic flux—the rate at which autophagosomes sequester and degrade cellular components—remain largely underexplored in humans during prolonged fasting. A key barrier to understanding human autophagy has been the reliance on cell culture methods that disrupt the natural nutritional and endocrine signalling present in blood. By maintaining cells in whole blood, which is physiologically intact during flux measurement, we aim to capture how autophagic flux responds to interventions like fasting while preserving the genetic, nutritional, and hormonal factors unique to each individual [12].

Other critical questions remain regarding fasting, including the optimal duration needed to trigger autophagy and whether exercise-induced glycogen depletion accelerates and potentiates this process, given glycogen’s role as the main glucose storage form in the human body. Moreover, while it is well established that prolonged fasting—unlike chronic caloric restriction—reduces circulating IGF-1 levels in humans [13,14,15,16], the effects of prolonged fasting on pathways such as insulin/IGF1/mTOR, which are crucial for DNA repair, cell proliferation, cancer suppression, and ageing, have not been thoroughly investigated in human studies [8,17,18,19]. Further research is essential to clarify how fasting activates cellular processes such as antioxidant defences, DNA repair, proteostasis, mitophagy, and cellular senescence inhibition, as well as its effects on telomere attrition and the modification of DNA methylation and proteomic ageing clocks in healthy individuals [20,21,22,23,24]. A deeper understanding of these mechanisms is crucial for translating the benefits of fasting into safe, therapeutic interventions grounded in well-defined biological pathways. The recent advancements in multi-omic technologies—including genomics, transcriptomics, epigenomics, proteomics, metabolomics, and microbiome metagenomics—offer an unprecedented opportunity to investigate the complex, multi-layered biological processes activated during fasting. These technologies enable the comprehensive profiling of molecular changes across various biological systems, allowing for the integration of data from different omics layers to reveal insights into metabolic regulation, autophagy, immune modulation, and cellular stress responses [25]. This systems-level approach can uncover intricate interactions that were previously inaccessible, potentially leading to the development of more targeted and personalised therapeutic strategies based on fasting-induced molecular alterations.

## 2. Aims of Profasta Trial

The primary aim of the PROFASTA trial is to evaluate the impact of a three-day water-only fast, with and without a single session of glycogen-depleting exercise, on autophagic flux in isolated peripheral blood mononuclear cells (PBMCs) where flux is measured by the introduction of a lysosomal inhibitor into whole blood. The primary hypothesis is that glycogen depletion through exercise prior to fasting will significantly enhance autophagic activation in PBMCs. Secondary objectives include assessing changes in body composition, heart rate variability, endothelial function (measured via flow-mediated vasodilation), as well as plasma metabolomic and proteomic shifts, and microbiome metagenomic adaptations in stool samples.

## 3. Methods

### 3.1. Study Design

**Figure 1:**
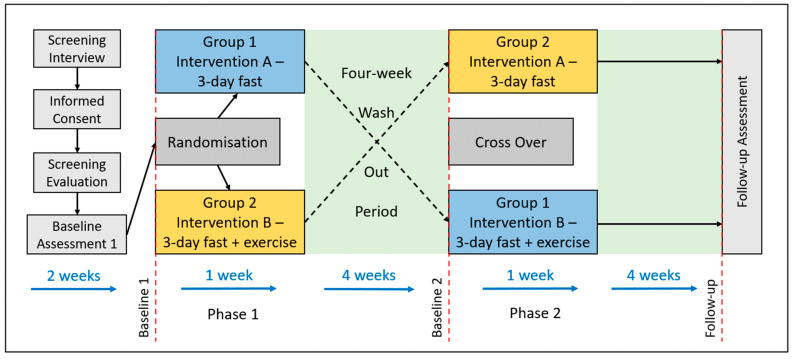
Experimental Design of the PROFASTA Trial. This randomised, two-period, two-sequence crossover trial involves 20 participants who are assigned to two interventions: Intervention A consists of a 3-day water-only fast, while Intervention B involves a 3-day water-only fast preceded by a single bout of glycogen-depleting endurance exercise performed on the first day of fasting. Each participant will complete both interventions, allowing for a comprehensive assessment of the effects of fasting and exercise on autophagic flux and other metabolic adaptations.

The PROFASTA trial is a randomised, single-centre, two-period, two-sequence crossover study, involving a total sample of 25 participants. Participants will be randomly assigned into two groups, with each group undergoing two different interventions in separate phases (Figure 1). By the study’s end, all participants will have completed both Intervention A and Intervention B, ensuring robust within-subject comparisons to strengthen the validity of the findings. Intervention A consists of a three-day water-only fast, while Intervention B includes a three-day water-only fast that is preceded by a single session of glycogen-depleting endurance exercise conducted on the first day of the fasting period, following an overnight fast. Detailed clinical visits and procedures are outlined in Table 1 and Table 2. This study has been approved by the Sydney Local Health District Ethics Committee (2022/ETH00617), and all study visits will take place at the Charles Perkins Centre-Royal Prince Alfred Hospital (CPC-RPA) clinic. The protocol has been prepared using the SPIRIT reporting guidelines [26].

### 3.2. Eligibility Criteria

Participants must meet the following criteria: (1) healthy males and females without disabilities that may hinder cycling exercise participation; (2) aged between 18 and 70 years old; (3) with a BMI within the range of 20–40 kg/m^2^; (4) able to adhere to all study assessments and procedures, including exercise and fasting interventions. Participants will be excluded based on the following conditions: (1) pregnant, breastfeeding, or of childbearing potential and unwilling to implement contraception during the study; (2) a history of chronic diseases that could impede exercise tests or compromise result interpretation; (3) the presence of comorbidities including hypertension, diabetes mellitus, or known cardiovascular disease; (4) a history of supplement or medication use that may affect result interpretation; (5) health conditions, dietary requirements, or psychosocial factors that may interfere with study participation and compliance; (6) unintentional weight loss exceeding 10% of body weight in the past 5 years, as well as smoking, psychiatric, or behavioural issues (e.g., a history of drug/alcohol abuse or eating disorders); and (7) the inability to perform cycling exercises, evidenced by an inability to complete the incremental peak power test during the baseline assessment or the 90 min glycogen-depleting exercise at the beginning of the fasting intervention (Table 3).

### 3.3. Recruitment and Initial Screening

Participants will be recruited through online platforms, including websites, emails, social media, and through flyers on university and hospital campuses. Interested individuals will be contacted by the study coordinator via email to complete an eligibility questionnaire and invited to an online screening visit. During this visit, they will receive comprehensive information about the study’s objectives, tests, and associated benefits and risks. Those who meet the inclusion criteria and are willing to participate will sign the consent form in person before commencing the screening evaluation visit (visit 1).

### 3.4. Baseline Visit

Consenting participants will be enrolled in the trial and undergo baseline clinical assessments, as outlined in Table 1 and Table 2. Following an overnight fast, participants will be screened by collecting medical, dietary, supplementation, and exercise history information and performing the Adult Pre-Exercise Screening System (APSS, 2019, version 2.0) assessment. Baseline evaluations will include the anthropometric measurements; collection of blood, urine, and stool samples; a buccal mucosa swab; an incremental VO_2_peak test on a cycle ergometer; and the completion of a wellbeing and symptoms questionnaire. Research volunteers will also receive instructions from a registered dietitian and study personnel for collecting a 10-day weighed food diary and adhering to fasting protocols. Continuous glucose monitor (CGM) along with a Fitbit device to monitor physical activity and sleep quality and duration will be also set up at baseline assessment.

**Table 1:** Summary of the visit schedule.

Study Phase	Visit	Visit Title	Approximate Duration of the Visit	Notes
Screening	1	Screening evaluation	3 h	Medical history, blood sample, VO_2_peak test
Phase 1	2	Baseline assessment 1	2 h	Clinical measurements
	3	Start of fasting and cycling exercise/rest period	3.5 h	Fasting starts a night before
	4	End of fasting 1	2 h	Refeeding starts after this visit
	5	End of refeeding 1	2.5 h	4 days after of end of fasting visit
Phase 2	6	Baseline assessment 2	2 h	4 weeks after visit 4
	7	Start of fasting and cycling exercise/rest period	3.5 h	Fasting starts a night before
	8	End of fasting 2	2 h	Refeeding starts after this visit
	9	End of refeeding 2	2.5 h	4 days after of end of fasting visit
	10	Follow up assessment	4 h	4 weeks after visit 8

### 3.5. Randomisation

Participants will be randomly assigned using a web-based system in REDCap, employing random permuted blocks with a block size of four. Randomisation will occur at the beginning of visit 3, after all baseline measurements have been completed (Figure 1, Table 1). The study coordinator will disclose group assignments to both the study team and the participant upon their arrival for the clinic visit #3. While blinding participants and investigators is not feasible due to the nature of the intervention, those conducting sample analysis and statistical analysis will remain blinded to group allocations, when possible.

### 3.6. Follow-Up Assessments

The study protocol includes a total of 10 visits, encompassing both baseline assessments and follow-up evaluations. Table 2 outlines the schedule of follow-up visits along with the corresponding testing procedures for the study.

**Table 2:** Schedule of visits and testing procedures at the CPC-RPA clinic.

		Phase 1				Phase 2				
Visit	1	2	3	4	5	6	7	8	9	10
	Screening Evaluation	Baseline Assessment 1	Start of Fasting and Exercise	End of Fasting 1	End of Refeeding 1	Baseline Assessment 2	Start of Fasting and Exercise 2	End of Fasting 2	End of Refeeding 2	Follow Up Assessment
Visit duration	3 h	2 h	3.5 h	2 h	2.5 h	2 h	3.5 h	2 h	2.5 h	4 h
Consent	☒									
Full body examination (BP, weight, waist/hip cm, vital signs)	☒	☒	☒	☒	☒	☒	☒	☒	☒	☒
Medical history	☒					☒				
Psychosocial questionnaires		☒		☒		☒		☒		☒
Inclusion/Exclusion		☒								
Randomisation			☒							
Fitbit wearable	☒	☒	☒	☒	☒	☒	☒	☒	☒	☒
Food diary		☒	☒	☒	☒	☒	☒	☒	☒	
Urine and faeces sample		☒		☒	☒	☒		☒	☒	☒
Exercise test/intervention	☒		☒				☒			
Fasting blood sample	☒		☒	☒	☒		☒	☒	☒	☒
Capillary glucose and ketones	☒	☒	☒	☒	☒	☒	☒	☒	☒	☒
CGM		☒				☒				
OGTT	☒									☒
DXA		☒								☒
Bodpod		☒	☒	☒	☒	☒	☒	☒	☒	☒
FMD			☒	☒	☒		☒	☒	☒	☒
Autonomic function (ECG)		☒		☒	☒	☒		☒	☒	☒
Buccal mucosa brush sample		☒	☒		☒		☒		☒	☒
Wellbeing/symptoms questionnaire		☒	☒	☒	☒	☒	☒	☒	☒	☒
Adverse Event Assessment	☒	☒	☒	☒	☒	☒	☒	☒	☒	☒

Legend: BP, blood pressure; CGM, continuous glucose monitor, DXA, dual-energy X-ray absorptiometry; ECG, electrocardiogram; FMD, flow-mediated dilation; OGTT, oral glucose tolerance test.

### 3.7. Intervention

**Figure 2:**
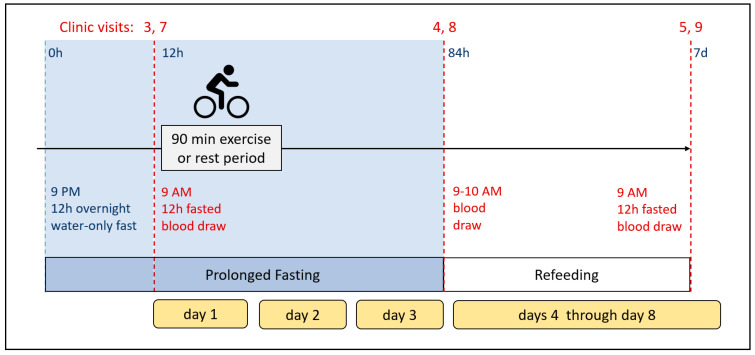
Intervention week and sequence of blood sample collection. This diagram outlines the schedule for blood sample collection during the intervention week (clinic visits 3–5 and 7–9). The intervention week includes the start of fasting, the end of fasting, and the end of refeeding visits and does not include the baseline visits or the final follow-up visit.

After completing the baseline assessments, participants will be randomly assigned to one of two groups: (1) Group 1 will undergo a prolonged water-only fast (Intervention A), while Group 2 will engage in a prolonged water-only fast accompanied by a single session of glycogen-lowering endurance exercise (Intervention B) during phase 1. Following this phase and a 4-week washout period, the groups will switch interventions in phase 2, with Group 1 undergoing Intervention B and Group 2 undergoing Intervention A. For a graphical representation of the experimental design, refer to Figure 1 and Figure 2.

**Table 3:** Eligibility Criteria for the PROFASTA Trial.

Inclusion Criteria	Exclusion Criteria
• Healthy males and females without disabilities affecting cycling ability	• Pregnancy, breastfeeding, or unwillingness to use contraception if of childbearing potential
• Age 18–70 years	• History of chronic diseases affecting exercise capacity or result interpretation
• BMI 20–40 kg/m^2^	• Presence of comorbidities (hypertension, diabetes, cardiovascular disease)
• Ability to comply with all study procedures and assessments	• Current use of supplements or medications that may affect results
	• Health conditions, dietary requirements, or psychosocial factors affecting compliance
	• Unintentional weight loss >10% of body weight in past 5 years
	• Current smoking, psychiatric conditions, or history of substance abuse/eating disorders
	• Inability to complete baseline exercise tests (incremental peak power test or 90-min glycogen-depleting exercise)

Note: All criteria must be met for study participation. BMI, body mass index.

## 4. Fasting and Exercise Interventions

### 4.1. Three-Day Fasting Protocol

Participants will undergo a three-day (approximately 84 h, including 12 h overnight fasting before clinic visit) water-only fast at home, with remote monitoring and support from the study team. Throughout the fasting and refeeding phases, participants will report their wellbeing and symptoms via an online questionnaire and can contact study coordinators through email, SMS, or phone. Adherence will be monitored using a continuous glucose monitor (CGM) and by measuring morning and evening beta-hydroxybutyrate (BHB) ketone and glucose levels through fingerpick capillary blood. Participants will communicate their glucose and ketones readings to the trial coordinator, who will also review the data stored in the device during clinic visits. The CGM data will be streamed to an online dashboard monitored by research staff.

A week prior to the fasting intervention, participants will transition to consuming only water for hydration to reduce dependence on caffeine and caloric beverages. They will refrain from strenuous exercise for 48 h before the fasting assessment. On the day before fasting begins, participants will consume only water from 9 PM onward and record all food and drink intake in a diary for submission after the refeeding assessments. During fasting, participants are advised to drink 2–3 litres of water daily, with unflavoured carbonated and mineral water permitted. If participants find it too challenging to continue water-only fasting, they will be provided with 250 mL of vegetable broth sourced from Undivided Food Co., Sydney, Australia, which contains 110 kJ of energy, 0.8 g of protein, 0 g of fat, 5.8 g of carbohydrate and 375 mg of sodium per 250 mL serving. A maximum of two servings per day will be provided. Light physical activities such as walking, yoga, or stretching are encouraged, while strenuous exercise is not recommended. On the last fasting day, following clinic assessments, food will be gradually reintroduced, starting with vegetable broth and transitioning to a low-carbohydrate vegetarian diet. This initial diet will consist of small portions of non-starchy vegetables, salads, and soups for the first day, then gradually transitioning to their normal diet by the end of the refeeding period. This gradual reintroduction of food aims to minimise potential adverse effects and gastrointestinal symptoms. The vegetable broth will be offered upon completion of the fasting visit, along with instructions for the subsequent four days of the refeeding period. The rest of the meals of the refeeding period will not be provided; however, the participants will log all the food and drink into the food diary. Once the refeeding period concludes, participants will be instructed to resume their usual lifestyle for a 4-week washout period.

### 4.2. Exercise-Induced Glycogen Depletion (80–90 Min)

The exercise protocol, adapted from seminal glycogen depletion studies [27,28], will take place after participants arrive for the start of the fasting visit following an overnight fast and after all initial assessments are complete. Power outputs for the cycle ergometer will be determined based on the incremental VO_2_peak test performed during the screening visit. Participants will engage in 60 min of cycling at a constant power output corresponding to 60–70% of their peak VO_2_, maintaining a cadence of 60–70 rpm, which is classified as moderate-intensity physical activity. After a 5 min rest, the participants will perform repeated 1 min sprints at 90 rpm and 90–100% of their VO_2_peak equivalent power output, with 1 min rest intervals between sprints. The exercise session will conclude after ten sprints or if participants cannot maintain a pedalling rate of ≥80–90 rpm for 1 min. Verbal encouragement will be provided throughout the session, during which heart rates will be recorded using Polar H10 chest strap heart rate monitors. Throughout the exercise session, participants’ fatigue levels will be monitored using the Rating of Perceived Exertion (6–20 RPE) scale, with heart rate and RPE recorded every 10 min during steady-state cycling and after each sprint. The exercise will be terminated if participants report severe fatigue (RPE ≥ 19), experience any adverse symptoms (dizziness, nausea, unusual shortness of breath), or if heart rate exceeds 95% of age-predicted maximum for more than 3 min. If a participant cannot complete the full protocol, a minimum of 60 min of steady-state cycling plus three sprints will be considered sufficient for partial glycogen depletion, allowing continued participation in the study. Those unable to meet these minimum requirements will be withdrawn from this arm of the trial.

## 5. Outcomes

### 5.1. Primary Outcome

The primary outcome will be the measurement of autophagy activation in isolated PBMCs from healthy individuals who have undergone a three-day water-only fast, with or without a single session of glycogen-depleting exercise using the methodology described previously [29].

### 5.2. Secondary Outcomes

Secondary outcomes to be assessed include:

- Body weight, waist circumference, and body composition measured by DXA and Bod Pod;
- Autonomic function, including heart age and heart rate variability, assessed through resting electrocardiography;
- Blood pressure;
- Endothelial function evaluated by flow-mediated vasodilation;
- Continuous monitoring of blood glucose kinetics;
- Capillary ketones and glucose levels using a portable test strip-based meter;
- Oral glucose tolerance test to assess glucose tolerance and insulin action;
- Serum fasting free fatty acids, BHB, acetoacetate, BHB-to-acetoacetate ratio, and lipid profile;
- Serum concentration of insulin, IGF-1, IGFBP-1, IGFBP-2, IGFBP-3, FGF21, cortisol, testosterone, estradiol, sex hormone-binding globulin (SHGB), T3, Klotho, BDNF, and Beta-Amyloids (Aβ40 and Aβ42);
- Markers of inflammation, including high-sensitivity C-reactive protein, cytokines (IL-6, TNF-alpha), and chemokines (MCP1);
- Markers of oxidative stress, including urinary F-2 isoprostanes and 8-hydroxy-2′-deoxy Guanosine.

### 5.3. Exploratory Outcomes

Several exploratory outcomes will also be examined:

- Levels of autophagy cargo receptor proteins in PBMCs such as p62/SQSTM1, NBR1, and OPTN;
- Calculated adjusted autophagy score based on (a) the primary outcome, (b) changes in PBMC pool make up, and (c) pre-existing data from cell type-specific autophagy calculated using flow cytometry;
- Plasma proteomics using Olink’s Proximity Extension Assay (PEA) technology to detect over 5400 proteins;
- Mass spectrometry-based proteomics and metabolomics of PBMCs;
- Plasma lipidomics and metabolomics;
- Detailed profiling of circulating immune cell populations and their functional states, along with molecular markers assessed via flow cytometry, including the lymphoid/myeloid ratio and a marker of protein acetylation in B cells;
- DNA methylation and genomic analysis in isolated PBMCs and buccal mucosa cells, including methylation-based ageing biomarkers such as DunedinPACE and GrimAge;
- Platelet characterisation involving proteomic analysis, flow cytometry, and activation assays to elucidate biochemical composition, surface marker expression, and functional responsiveness;
- Characterisation of plasma extracellular vesicles (EVs) using nanoparticle tracking analysis, transmission electron microscopy, and the proteomics of isolated EVs to determine size distribution, morphology, and cargo composition;
- Analysis of the gut microbiome through 16S ribosomal RNA (rRNA) gene amplicons and shotgun metagenomics.

### 5.4. Safety Monotoring and Reporting

Participant safety is prioritised through comprehensive screening, monitoring, and risk mitigation strategies. Screening excludes individuals with conditions that could increase risk, including a history of hypoglycemic episodes, cardiovascular problems, or eating disorders. During the fasting period, participants are monitored via continuous glucose monitoring, additional twice-daily glucose and ketone measurements, and regular check-ins with study staff. Medical supervision is provided during exercise sessions, with clear stopping criteria based on heart rate, blood pressure, and symptoms. Emergency protocols include immediate exercise termination criteria and breaking-fast procedures if participants experience severe adverse effects. Adverse events (AE) and serious adverse events (SAE) will be systematically documented and reported throughout the study to prioritise participant safety. Each event will include details such as a description, onset date, end date, severity, assessment of relatedness to the trial intervention or device, and any actions taken in response. Research investigators will use their clinical expertise to determine whether an AE necessitates the participant’s removal from the study. SAEs will be reported to the primary investigator within 24 h of the study team’s awareness. The complete course of the SAE, including any administered therapy, will be communicated to the study sponsor. Additionally, any significant safety concerns will be reported to the local Human Research Ethics Committee and Research Governance Officers.

### 5.5. Sample Size Calculation

A total of 25 volunteers will be recruited for the study. Given the crossover design, each participant will undergo both interventions, effectively creating two groups of 25. To estimate the required sample size, the change in LC3B-II to beta-actin ratio was considered. LC3B-II, the lipidated form of microtubule associated protein 1 light chain 3 beta that accumulates on autophagosomes when lysosomal degradation is blocked, is the gold standard marker for measuring autophagic flux in human samples [30]. Utilising ANOVA with a repeated measures design, assuming a standard deviation of 95.4, and aiming for 80% power at a significance level of 0.05, a difference of 30% (a change of 134.7 from a mean of 299.3 ng/mg protein/hour in the control group at baseline) could be detected with a sample size of 20 per group. Accounting for an estimated dropout rate of 20%, the initial recruitment target is set at 25 participants, based on the study by Bensalem et al. [29]. Sample size versus power plot is shown in Supplementary Figure S1. Additionally, sensitivity analyses, examining sample size requirements for detecting 20% and 40% changes, are shown. The exploratory outcomes will not be separately powered but will provide valuable hypothesis-generating data for future targeted studies.

### 5.6. Statistical Analysis Plan

Statistical analysis will employ ANOVA with a repeated measures design and/or linear mixed model regression. The models will include baseline characteristics such as age, sex, prior fasting experience, and habitual dietary patterns as covariates. While our sample size is primarily powered for detecting changes in the primary outcome, we will conduct exploratory analyses of these potential modifying factors, acknowledging that subgroup analyses will be hypothesis-generating rather than definitive. Linear regression and/or general additive models will analyse the relationship between habitual dietary intake and baseline measures, as well as the magnitude of response to fasting. Additionally, correlation analyses will be conducted to evaluate the relationship between autophagic flux readouts and quantified molecular markers, aiming to identify potential biomarker candidates for future studies. Given the sample size, analyses stratified by factors such as sex, age groups, or physical activity levels will be considered exploratory and interpreted with appropriate caution.

Exploratory multi-omics outcomes will be integrated via a multi-step approach. First, each omics layer (e.g., metabolomics, proteomics, or transcriptomics) will be analysed independently using standard workflows. Integration will then proceed through: (1) correlation network analysis to identify associations between molecules across omics layers; (2) pathway enrichment analysis to uncover biological processes affected across these layers; and (3) machine learning approaches, including sparse partial least squares discriminant analysis (sPLS-DA), to identify key molecular signatures associated with changes in autophagic flux. Specifically, metabolomics data will identify altered metabolic pathways, which will be cross-referenced with proteomic changes in related enzymes and regulatory proteins. Additionally, changes in the gut microbiome will be correlated with plasma metabolites to identify potential microbe-derived metabolites. This integrated analysis aims to provide a systems-level understanding of fasting-induced molecular changes and their relationship to autophagic flux. Established multi-omics integration tools such as mixOmics and MOFA+ (Multi-Omics Factor Analysis) will be employed for the analysis.

### 5.7. Ethics Statement

The study will adhere to the National Statement on Ethical Conduct in Human Research (2007), the CPMP/ICH Note for Guidance on Good Clinical Practice, and the principles of the Declaration of Helsinki. Compliance with these standards ensures that the rights, safety, and wellbeing of participants are protected. This study has received approval from the Sydney Local Health District Ethics Committee (2022/ETH00617). All participants will provide written informed consent.

## 6. Discussion

The PROFASTA trial aims to comprehensively evaluate the effects of a combined approach of prolonged fasting and glycogen-depleting exercise on autophagy activation and stress-resistance pathways in humans. This investigation is both timely and crucial, given the growing interest in fasting as a potential strategy for promoting healthy longevity and mitigating the risk of age-related diseases, independent of dietary restriction with optimal nutrient intake or regular moderate-intensity exercise training.

Preclinical data and various non-randomised clinical studies suggest that prolonged fasting in humans may provide numerous health benefits beyond simple weight loss; however, it may also carry potential risks [1,9,31]. These risks include headache, dizziness, nausea, fatigue, muscle weakness, and low blood pressure during fasting and refeeding, as well as exercise-related risks such as excessive fatigue and muscle soreness during glycogen-depleting protocols. However, careful screening, monitoring, and safety protocols can effectively manage these risks in healthy participants. The potential benefits include improved metabolic health, enhanced insulin sensitivity, reduced inflammation, and the induction of autophagy, along with other molecular changes in critical ageing pathways that can positively influence overall healthspan and lifespan [32]. However, rigorous randomised clinical trials have yet to test these hypotheses using comprehensive study designs that integrate multiple molecular readouts, state-of-the-art autophagy flux methodologies, and real-time continuous glucose monitoring sensors.

Fasting for several days can be extremely challenging, so any intervention that could accelerate or potentiate this process would be beneficial. Ketosis typically begins when glycogen stores become depleted and cannot provide sufficient glucose for the brain, which cannot utilise lipids or proteins for energy [3]. Intense exercise can rapidly deplete glycogen stores, particularly in muscle tissue, as glycogen is the primary fuel source during prolonged or high-intensity physical activity [33,34,35]. During high-intensity, intermittent endurance exercise (above 75% VO_2_peak), marked reductions in glycogen stores occur within 30–60 min [36,37]. This is because such intense activity places a high energy demand, causing muscles to rely heavily on glycogen as a primary fuel source. This depletion forces the body to shift towards alternative energy sources, such as fatty acids and ketone bodies, which can accelerate metabolic adaptations like ketosis. Our primary hypothesis is that glycogen depletion through exercise prior to fasting will significantly amplify autophagy activation and other molecular pathways that slow ageing processes, compared to fasting alone.

Autophagy is a crucial cellular process that maintains homeostasis by degrading and recycling damaged cellular components. It has emerged as a key mechanism associated with various health benefits linked to calorie restriction and fasting, including mitophagy and improved mitochondrial function, increased stress resistance, and reduced cellular senescence [23,38]. Through the investigation of autophagic flux and related molecular adaptations, the PROFASTA trial aims to elucidate the precise mechanisms by which fasting influences cellular quality control processes in humans. In addition to assessing autophagy, this trial will explore potential biomarkers for autophagic activity using advanced multi-omics approaches, including genomic, epigenomic, proteomic, and metabolomic analyses. Identifying reliable biomarkers is crucial for advancing our understanding of fasting’s biological effects and developing targeted therapeutic interventions. For instance, the recent literature highlights the role of specific proteins and metabolites as indicators of autophagic activity, which can inform future clinical trials aimed at testing dietary interventions, such as fasting and ketogenic diets [30]. While we prioritised non-invasive blood-based measurements to optimise participant retention in this initial study, future investigations will need to examine tissue-specific autophagy activation and establish its correlation with whole blood measurements.

The implications of our findings extend beyond an individual’s health. With ageing populations globally, understanding how lifestyle interventions, including fasting, can promote cellular health and promote long-term health is paramount [39]. The PROFASTA trial could inform evidence-based recommendations for healthcare providers by establishing safety parameters, optimal timing, and monitoring requirements for combined fasting–exercise protocols. These findings may help develop practical guidelines for implementing fasting interventions in clinical settings and lifestyle programmes. Furthermore, this research aligns with growing evidence that emphasises the importance of personalised approaches to health interventions. Individual variations in metabolic responses to fasting and exercise highlight the need for tailored strategies that consider sex, age, genetic and epigenetic background, and lifestyle factors. By integrating insights from our study with the existing literature, we aim to contribute to the development of targeted fasting protocols that can be safely implemented across diverse populations to promote cellular and metabolic health.

## 7. Conclusions

In conclusion, the PROFASTA trial represents a critical step toward elucidating the interplay between fasting, exercise, autophagy, and ageing. By establishing safe and effective interventions that stimulate autophagy and other anti-ageing mechanisms, we can advance the field of health promotion and disease treatment research, paving the way for future studies that explore the full potential of fasting as a therapeutic modality. As we continue to uncover the biological underpinnings of fasting and its health benefits, we can foster a deeper appreciation of the role of dietary and lifestyle choices in enhancing healthspan and quality of life.

## Data Availability

No new data were created or analysed in this study. Data sharing is not applicable to this article.

## Supplementary Materials

The following supporting information can be downloaded at: https://www.mdpi.com/article/10.3390/nu16244297/s1, Figure S1: Sample Size Sensitivity Analysis for Detecting Changes in Autophagic Flux.
