# Supplementary material for: Investigating the Impact of Glycogen-Depleting Exercise Combined with Prolonged Fasting on Autophagy and Cellular Health in Humans: A Randomised Controlled Crossover Trial
Source: Nutrients. 2024 Dec 12;16(24):4297. doi: 10.3390/nu16244297 (PMC11677747; doi:10.3390/nu16244297)
Supplement: Supplementary file 1 [file nutrients-16-04297-s001.zip › nutrients-3334755-supplementary.pdf]

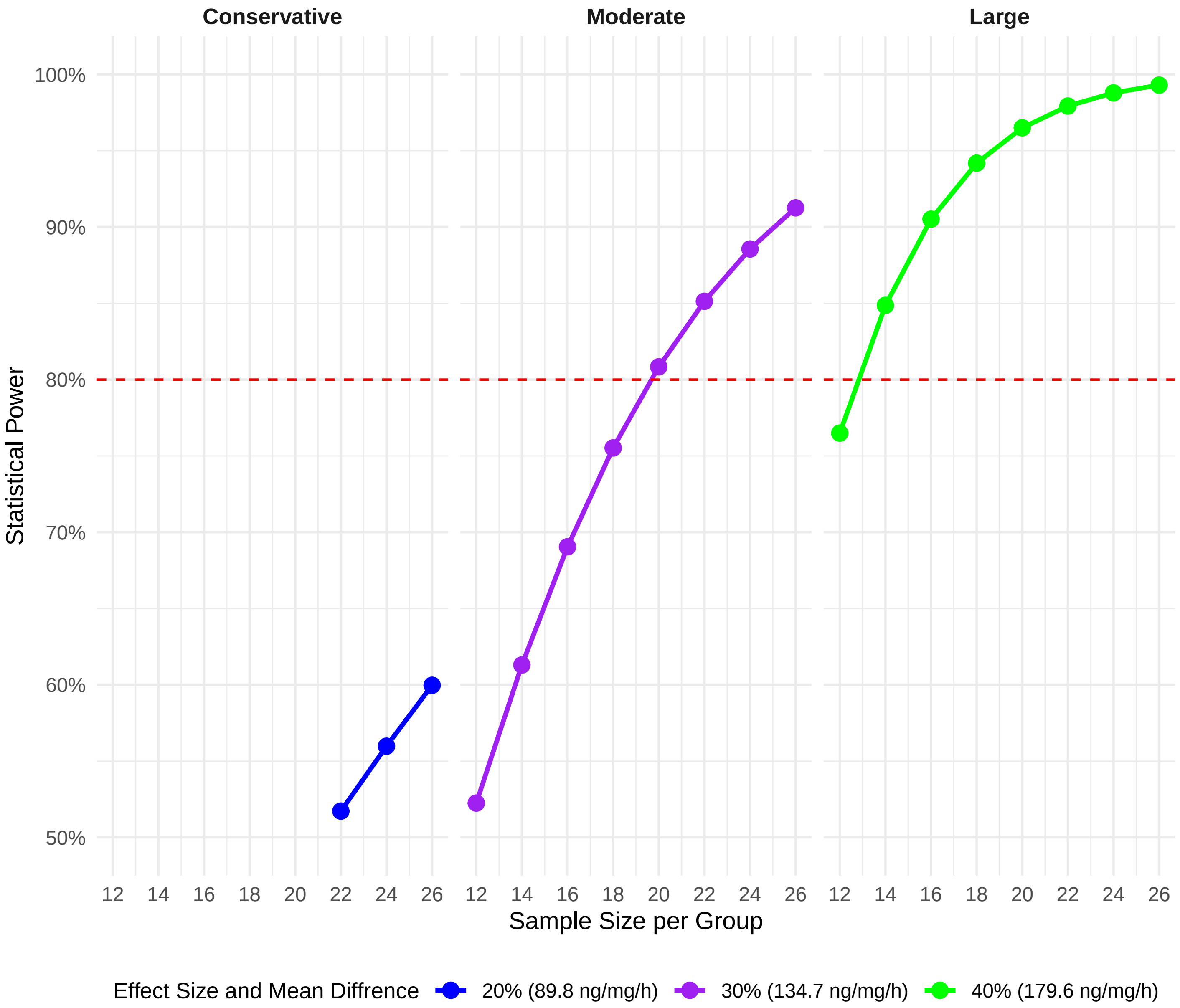

Supplementary Figure 1. Sample Size Sensitivity Analysis for Detecting Changes in Autophagic Flux. Power calculations for repeated measures ANOVA showing required sample sizes to detect different magnitudes of change in autophagic flux, measured as LC3B-II to beta-actin ratio (baseline: 299.3 ± 95.4 ng/mg protein/hour). Three effect sizes are presented: 20% or 89.8 ng/mg protein/hour (blue), 30% or 134.7 ng/mg protein/hour (purple), and 40% or 179.6 ng/mg protein/hour (green). Calculations assume 80% power and α=0.05. The selected sample size of 20 participants provides adequate power to detect a 30% change in autophagic flux, with greater power for larger effects and reduced power for smaller changes.
